# When Random Variation Results in Functional Segregation

**DOI:** 10.1007/s12021-026-09779-0

**Published:** 2026-05-19

**Authors:** Jacob Barfield, Patrick Kells, Shree Gautam, Woodrow Shew

**Affiliations:** 1 Physics Department, Hollins University, 7916 Williamson Rd, Roanoke, VA 24020 USA; 2 Physics Department, University of Arkansas, 1 University of Arkansas, Fayetteville, AR 72701 USA

**Keywords:** Functional segregation, Motor cortex, Heavy-tailed distributions

## Abstract

Many functional properties vary dramatically across neurons in cerebral cortex. Two fundamental goals of systems neuroscience are to determine which neurons execute which functions and how the different functional properties of a neuron are related. Here we focus on *functional segregation* - when two separate subpopulation of neurons encodes two distinct functions. Often, it is assumed that if two functional properties are uncorrelated across the population, then there is no functional segregation. Here we show that this assumption can lead to wrong conclusions; functional segregation can emerge, by chance, due to random variation when that variation is distributed according to skewed, heavy-tailed distributions. We reexamine the results we previously reported (*Nature communications*, *10*(1), 1575–1575 2019), which showed that neurons in primary motor cortex tend to be functionally segregated, with neurons that are either strongly coupled to body movements or to ongoing cortical population activity. Here we show that this is a prime example of functional segregation due to random variation.

**Supplementary Information:**

The online version contains supplementary material available at 10.1007/s12021-026-09779-0.

## Introduction

As technological innovations push towards recordings of larger and larger populations of neurons (e.g. Stringer et al. (2019); Ponce-Alvarez et al. (2018)), it becomes feasible to assess similarities and differences across neurons with greater statistical rigor. Such assessments have revealed that many properties of neurons are not normally distributed across neurons (Buzsáki & Mizuseki, 2014). Firing rates (Fujisawa et al., 2008; Hromádka et al., 2008; Mizuseki & Buzsáki, 2013), synaptic strengths (Lefort et al., 2009; Mizuseki & Buzsáki, 2013; Song et al., 2005), pairwise spike correlations (Mizuseki & Buzsaki, 2014), dendritic spine size (Loewenstein et al., 2011), and axon calibers (Wang et al., 2008) are a few examples of properties that seem to be distributed according asymmetric, skewed distributions with heavy tails. This means extreme values, although not common, occur far more often than one would expect for normally distributed quantities.

One tradition in data analysis is to ignore the extreme values, assuming that there is something wrong with the data points that fall very far from the mean (Barnett, 1994; Zimek & Filzmoser, 2018). For instance, by default, Matlab’s box and whisker plot function defines “outliers" as any data point that lies more than 1.5 times the interquartile range above the upper quartile or below the lower quartile. However, for data that is distributed in a skewed non-Gaussian, heavy-tailed way, it is inappropriate to discard extreme values. Ignoring or mishandling heavy-tailed data can result in wrong interpretations (e.g. distorted model fitting Karlsson et al. (2024); Vogel et al. (2025) and estimating correlations Cohen et al. (2020)).

For example, considering synaptic efficacy; suppose we find that 1 in 1000 synapses is so strong that a single spike arriving at the presynaptic side can elicit an action potential from the post-synaptic neuron (Lefort et al., 2009; Mizuseki & Buzsáki, 2013; Song et al., 2005). Considering that each neuron has 1000s of afferent and efferent synapses, this implies a “backbone" network of strong synapses that can propogate signals very effectively without even considering the multitude of weaker, typical synapses (Yassin et al., 2010) similar to the “rich club" structure that has been observed at cellular scales (Nigam et al., 2016) and at macro scales (Heuvel & Sporns, 2011). Thus, in the case of synaptic efficacy, ignoring extreme values may miss the most important and reliable circuit for transmitting information.

In this paper, we address another interesting implication and potential mistake when dealing with long-tailed distributions of neuronal properties. The case we consider is motivated by our previous study of neurons in motor cortex of rats (Kells et al., 2019). In this study, spike activity recorded from about 1000 single neurons were analyzed to quantify two properties for each neuron. First, they measured “body coupling" for each neuron, which quantifies how strongly the neuron’s activity is correlated with body movement (Methods, Kells et al. (2019)). Second, they measured “population coupling", which quantifies how strongly the neuron’s activity is correlated with the ongoing activity of the cortical population in which it is embedded (Methods, (Kells et al., 2019; Okun et al., 2015). They discovered a weak anti-correlation between body and population coupling; neurons with high body coupling tended to have low population coupling, and vice versa. The weak anti-correlation was considered as weak, but significant, evidence that these two properties were functionally segregated. In this paper, we propose a new way to measure functional segregation that better accounts for the fact that body coupling and population coupling are both distributed in a way that is highly non-Gaussian, with heavy tails. Our proposed metric of functional segregation highlights the fact that such heavy-tail-distributed properties can be functionally segregated irrespective of how correlated the properties are.

## Materials and Methods

### Animals, Electrophysiology, and Body Motion Tracking

Experimental data from Kells et al. (2019) was reanalyzed in this study. All procedures were carried out in accordance with the recommendations in the Guide for the Care and Use of Laboratory Animals of the National Institutes of Health and approved by University of Arkansas Institutional Animal Care and Use Committee (protocol 14048). We studied adult male rats (n = 6 *Rattus norvegicus*, Sprague-Dawley outbred, Harlan Laboratories, TX, USA). We chronically implanted 32-channel electrode arrays in deep layers of motor cortex (600-1200 $\mu$m depth). We performed electrophysiological recordings (30 kHz sample rate) and spike sorting to obtain single-unit spiking activity (n = 1258 single units, n= 143 recordings, 30 min each). A nine-camera motion tracking system (Flex:V100R2, Naturalpoint) tracked the three-dimensional coordinates of eight reflective beads (MCP1125, Natural point, 3 mm diameter), adhered to the rat’s neck, back, rear hips, and base of tail. The camera frame rate was 100 Hz and the system measured the bead positions with sub-millimeter resolution. The rats were placed on a 30 cm $\times$ 30 cm square stage inside of a dark box and allowed to move freely, without constraint or trained task, during each 30 min recording.

### Experimental Data Analysis

As in previous studies (Kells et al., 2019; Okun et al., 2015), *Population coupling* was defined for each neuron based on its spike count time series.

1 $$\begin{aligned} C_{pop,i} = \frac{1}{N_{i}}\sum _{t=1}^{T} f_{i}(t)P_{i}(t) \end{aligned}$$

where $f_{i}(t)$ is the spike count in the $t^{th}$ time bin of neuron *i*, $N_{i}$ is the total number of spikes for neuron *i* over the whole recording. Time bins were chosen to be 0.25 sec in duration. The population spike count is defined as

2 $$\begin{aligned} P_{i}(t) = \sum _{j\ne i}(f_{j}(t) - \mu _{j}), \end{aligned}$$

where the mean spike count for neuron *j* is $\mu _{j}$. The population coupling is poorly estimated when there are only a small number of recorded neurons. This is why recordings with fewer than 5 units recorded were excluded from analysis.

As defined previously (Kells et al., 2019), *Body coupling* was measured in 2 different ways. Both methods were based of of the rat’s body movement and how strongly each recorded unit is related to this movement. Two different properties were used to determine these measures of body coupling. The first property was a movement-triggered-average spike rate (MTASR). First, movement onset events were defined as the moments when the rats’ body speed exceeded it’s mean value (the mean was very close to zero due to long periods of rest). Then the MTASR was calculated as the triggered-average spike-count time series in a $\pm 1 s$ window around the movement onset times (like a typical peri-stimulus time histogram (PSTH) analysis in experiments with a repeated stimulus). The value of body coupling $BC_{M}$ was defined as the standard deviation of the MTASR. The second property used to analyze body coupling is a spike-triggered-average body speed (STABS). This is the average of the body speed within a $\pm 1 s$ window of each time a neuron fires. The body coupling $BC_{S}$ is defined as the standard deviation of this STABS for each neuron. In both cases the waveform were low-pass filtered using a 1.5 Hz filter and normalized by their mean.

**Fig. 1:**
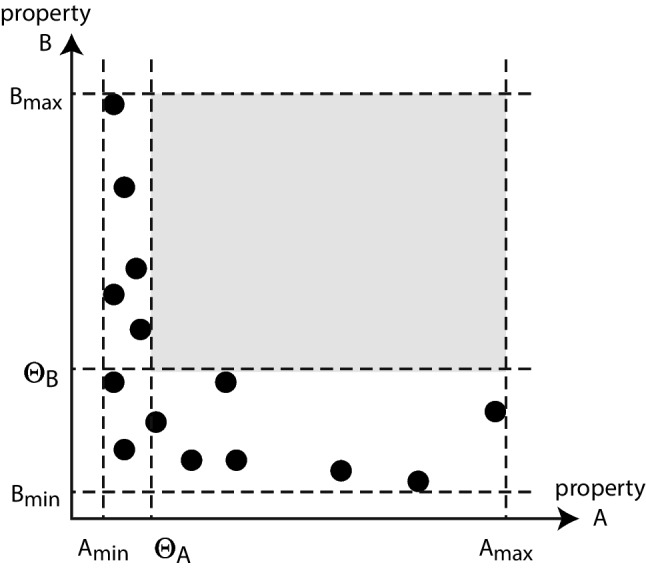
**Defining functional segregation metric** $\Sigma$. Each point represents one neuron. If two properties, **A** and **B**, are functionally segregated across the neural population, then by our definition, the neurons with high values of property **A** will have low values of property **B**, and vice versa. In this diagram, functional segregation implies that there is a large area (gray) in the upper right portion without any points. To quantify functional segregation, we consider all possible choices of two thresholds, $\Theta _A$ and $\Theta _B$, selecting values that maximize the shaded area. $\Sigma$ is defined as the ratio of the empty gray area to the larger area $(A_{max}-A_{min})(B_{max}-B_{min})$ for the choice of $\Theta _A$ and $\Theta _B$ that maximizes this ratio Eq. 3

### Functional Segregation $\Sigma$

For a given population of neurons with two functional properties measured for each neuron, let us call them A and B, we ask the question: Is the population functionally segregated into two sub-populations, one that does A and another that does B? In the specific example we work with, A will be body coupling and B will be population coupling. To answer this question we suggest a new metric $\Sigma$ to quantify functional segregation as defined in Eq 3. The rationale behind our definition is illustrated in Fig. 1; the idea is that if the population is functionally segregated, then neurons will either have property A or property B, but not both. This idea is quantified by $\Sigma$ as follows.

3 $$\begin{aligned} \Sigma = \underset{\Theta _A,\Theta _B}{{\text {max}}}\left( \frac{A_{max}-\Theta _A}{A_{max}-A_{min}} \cdot \frac{B_{max}-\Theta _B}{B_{max}-B_{min}}\right) \end{aligned}$$

**Fig. 2:**
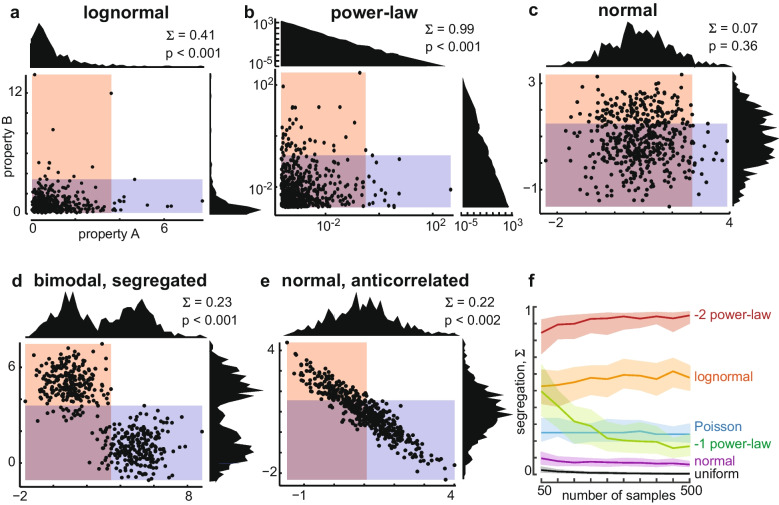
**Functional segregation from random distributions.** $\Sigma$ quantifies functional segregation by chance and in more traditional scenarios. (**a**-**e**) Each point in the scatter plot represents one neuron. The horizontal and vertical coordinates of each point are determined by values for two hypothetical properties of the neuron (properties A and B, respectively). Distributions of property A are shown above each scatter plot. Distributions of property B are shown to the right of each scatter plot. The red shaded area indicates the range of A that is less than $\Theta _{A}$. The blue shaded area delineates B less than $\Theta _{B}$. The neurons in the red area that does not overlap the blue area are selective for property B and functionally segregated from the neurons in the non-overlapping blue area, which are selective for property A. Our segregation measure $\Sigma$ quantifies the range of non-overlapping red and blue areas. Skewed distributions like the log-normal case (**a**) and the power-law (**b**) can exhibit significant $\Sigma$ even though properties A and B are uncorrelated. Note that the power-law distributions and scatter plot have logarithmic axes for ease of visualization. Uncorrelated normally distributed properties are not significantly segregated (quantifies functional segregation ). Anti-correlated properties also have significant segregation (**d**, **e**). **f**) The $\Sigma$ measure is insensitive to sample size for more than a few hundred samples

**Fig. 3:**
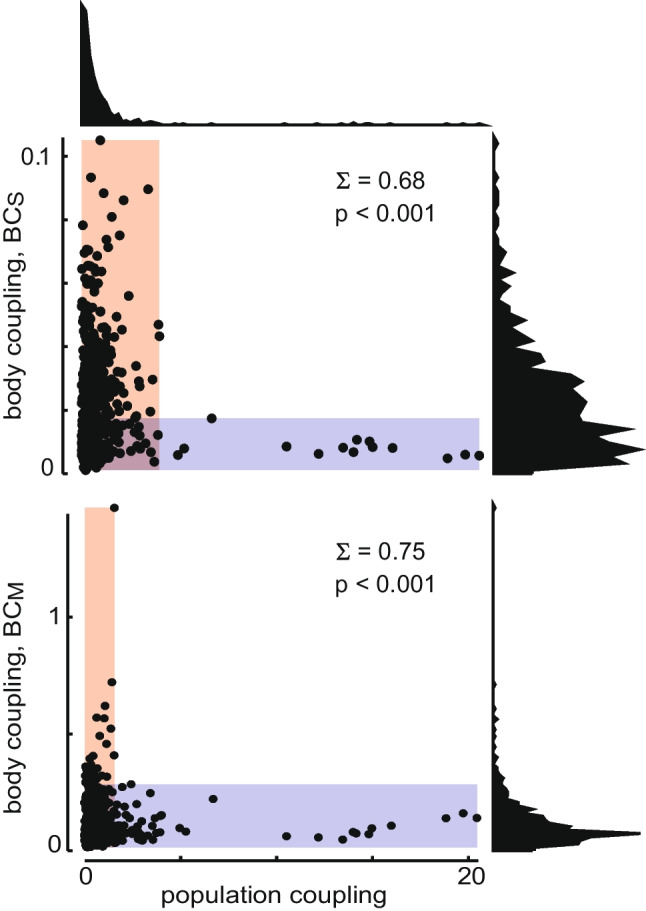
**Functional segregation of population coupling and body coupling in motor cortex.** Due to the heavy-tailed distributions of population coupling and body coupling, these two properties are strongly functionally segregated, even though these two properties are weakly correlated

In practice, to compute $\Sigma$, all possible pairs of thresholds $\Theta _A$ and $\Theta _B$ are tested and then chosen to maximize $\Sigma$ while meeting the constraint that no single neuron has $A > \Theta _{A}$ and $B > \Theta _{B}$. In other words, the thresholds are defined to maximize the area of the gray region without any points in Fig. 1. In the following results, the thresholds are the right and top boundaries, respectively, of the red and blue shaded areas in Figs. 2 and 3; the constraint requires that there are no neurons in the white area in the upper right section of the plots.

By definition $\Sigma$ will be have some value between 0 and 1; $\Sigma \approx 1$ indicates extreme functional segregation, while $\Sigma \approx 0$ indicates a lack of functional segregation. How do we decide if a given intermediate value of $\Sigma$ is significant? One way to approach this question is to compare to a case where we do not expect any meaningful functional segregation - when the two properties are drawn from a two-dimensional uncorrelated Gaussian. With this in mind, we define a p value, defined as the probability of the null hypothesis that the measured $\Sigma$ results from an equal number of samples drawn from a two-dimensional uncorrelated normal distribution with the same means and standard deviations as the measured values of A and B. To calculate this p value, we simply generate 1000 surrogate data sets drawn from the null hypothesis distribution, calculate a surrogate $\Sigma$ for each, and quantify the fraction of these that are greater than the actual $\Sigma$.

### Statistical Models

In Fig. 2, we examined how $\Sigma$ varies when the properties A and B were drawn from six different distributions. These included an uncorrelated two-dimensional normal distribution, two different uncorrelated power-law distributions (with exponents of -1 and -2), an uncorrelated log-normal distribution, a sum of two normal distributions with different means, an anti-corrleated two-dimensional normal distribution, and a Poisson distribution. to an anti-correlated bi-modal distribution. To numerically generate samples from these distributions, we used inverse transform sampling. For the summary results in Fig. 2f, each distribution was run using 100 different radnom number generator seeds for each number of samples. For power law distributions and the uniform distribution we implemented upper and lower limits to ensure that the inverse transformation process was possible. The lower limit was chosen to be 0.01 and the upper limit was chosen to be 100. These same distributions did not have a way to set the mean and standard deviation so we implemented a tolerance level of 0.1 meaning that the distribution that was generated must have a mean and standard deviation that is within 0.1 of the mean and standard deviation chosen for all of the rest of the distributions.

## Results and Discussion

Consider a population of N neurons and two functional properties, say A and B, measured for each neuron. For instance, these two properties could represent response to different sensory stimuli or genetic properties, or motor coding properties. When can we claim that two different properties are functionally segregated across the population, meaning that one subset of neurons does A and a separate subset of neurons does B? Clearly, A and B are not functionally segregated if they are uncorrelated and normally distributed (Fig. 2c). Some simple examples that clearly are functionally segregated are when the two properties are negatively correlated (Fig. 2e, d) or clustered (Fig. 2d). These three cases might suggest that functional segregation requires a non-random relationship between A and B, but this is incorrect. If A and B are distributed according to a heavy-tailed distribution, functional segregation can be strong ($\Sigma$ of order 1), even if A and B are totally uncorrelated. We show this for log-normal, power-law, and Poisson distributions in Figs. 2a, b, and f. In these cases, the A and B properties reach extreme values, but are unlikely to both be extreme for the same neuron, simply because the extreme values are improbable. Thus, for heavy-tail-distributed properties, we expect to find functionally segregated subpopulations of neurons, by chance, without any coordinating mechanism.

We found that as the number of samples used increases the accuracy with which a value for $\Sigma$ can be determined increases making the results more reliable (Fig. 2f). The results also show that for heavy tail distributions $\Sigma$ tends to increase slightly as the number of samples increases (with exception of the power law with exponent equal to -1). The distributions used that are not heavy tail all resulted in low values for functional segregation that remained relatively constant as the number of samples increased. We also found that beyond about 200 samples there was no significant change in the value for functional segregation as sample size increased.

Next, we will demonstrate an example of functional segregation due to heavy-tail-distributed properties in experimental data. In our previous study of rat motor cortex (Kells et al., 2019), which motivated our considerations here, we found that neurons in deep layers of rat primary motor cortex were functionally segregated, very similar to the scenario described here. The two properties we considered were population coupling and body coupling. The population coupling of a neuron quantifies how its firing co-varies with the firing rate of the population in which it is embedded (Kells et al., 2019; Okun et al., 2015). The body coupling of a neuron quantifies how much its firing co-varies with movements of the body (Kells et al., 2019). We found that neurons with high body coupling had low population coupling and neurons with high population coupling had low body coupling (Fig. 3a). This observation suggests that the neurons are functionally segregated into an "internal" group (those with strong population coupling) and an "external" group (those with strong body coupling). We did not observe overlap in these groups; there were no neurons with both very high body coupling and very high population coupling.

We reanalyzed these data using our functional segregation metric $\Sigma$ (Fig. 3). For both forms of body coupling there we find $\Sigma$ near 1 and strongly reject the null hypothesis that such a $\Sigma$ could result from normally distributed properties. This is significant because it confirms one of the main conclusions from the Kells et al. paper without relying on the use of correlation analysis. In the original paper this result relied on a weak peaked but statistically significant relationship between the two properties that showed a weak correlation between the two, but the properties were nearly uncorrelated. We emphasize that the lack of correlation does not imply a lack of functional segregation.

## Conclusions

In this paper we proposed a new method for analyzing functional segregation that is valid and useful when the functional properties in question are uncorrelated and unclustered. We re-analyzed properties of neurons measured in motor cortex of rats, showing that these neurons are functionally segregated with one population involved in body movement and a separate population invovled in internal ongoing cortical dynamics. Our observations of functional segregation by chance is reminiscent of similar ideas in which a randomly wired network of neurons can do quite useful things. This idea manifests in liquid state computing (Maass et al., 2002) and the ’pre-configured brain’ concept proposed by Buzsaki and colleagues (Buzsáki & Mizuseki, 2014; Mizuseki & Buzsáki, 2013).

## Supplementary Information

Below is the link to the electronic supplementary material.

Supplementary file 1 (zip 10 KB)

## Data Availability

All Matlab files used for data generation and analysis are provided in the supplementary information files.

## References

[CR1] Barnett, V. (1994). Outliers in Statistical Data, Third edition. edn. Wiley series in probability and mathematical statistics. Applied probability and statistics. Wiley & Sons.

[CR2] Buzsáki, G., & Mizuseki, K. (2014). The log-dynamic brain: how skewed distributions affect network operations. *Nature Reviews Neuroscience,**15*(4), 264–278. 24569488 10.1038/nrn3687 PMC4051294

[CR3] Cohen, J. E., Davis, R. A., & Samorodnitsky, G. (2020). Heavy-tailed distributions, correlations, kurtosis and taylors law of fluctuation scaling. *Proceedings of the Royal Society A,**476*(2244), 20200610. 10.1098/rspa.2020.0610 PMC7776978 33408562

[CR4] Fujisawa, S., Amarasingham, A., Harrison, M. T., & Buzsáki, G. (2008). Behavior-dependent short-term assembly dynamics in the medial prefrontal cortex. *Nature Neuroscience,**11*, 823–833. 18516033 10.1038/nn.2134 PMC2562676

[CR5] Heuvel, M. P., & Sporns, O. (2011). Rich-club organization of the human connectome. *The Journal of Neuroscience,**31*(44), 15775–15786. 22049421 10.1523/JNEUROSCI.3539-11.2011 PMC6623027

[CR6] Hromádka, T., Deweese, M. R., & Zador, A. M. (2008). Sparse representation of sounds in the unanesthetized auditory cortex. *PLoS Biology,**6*(1), 16–16. 10.1371/journal.pbio.0060016 PMC2214813 18232737

[CR7] Karlsson, M., Wang, Y., & Ziebarth, N. R. (2024). Getting the right tail right: Modeling tails of health expenditure distributions. *Journal of Health Economics,**97*, Article 102912. 39013330 10.1016/j.jhealeco.2024.102912

[CR8] Kells, P. A., Gautam, S. H., Fakhraei, L., Li, J., & Shew, W. L. (2019). Strong neuron-to-body coupling implies weak neuron-to-neuron coupling in motor cortex. *Nature Communications,**10*(1), 1575–1575. 30952848 10.1038/s41467-019-09478-2 PMC6450901

[CR9] Lefort, S., Tomm, C., Sarria, J. C. F., & Petersen, C. C. H. (2009). The excitatory neuronal network of the c2 barrel column in mouse primary somatosensory cortex. *Neuron,**61*, 301–316. 19186171 10.1016/j.neuron.2008.12.020

[CR10] Loewenstein, Y., Kuras, A., & Rumpel, S. (2011). Multiplicative dynamics underlie the emergence of the log-normal distribution of spine sizes in the neocortex in vivo. *The Journal of Neuroscience,**31*(26), 9481–9488. 21715613 10.1523/JNEUROSCI.6130-10.2011 PMC6623170

[CR11] Maass, W., Natschläger, T., & Markram, H. (2002). Real-time computing without stable states: A new framework for neural computation based on perturbations. *Neural Computation,**14*(11), 2531–2560. 12433288 10.1162/089976602760407955

[CR12] Mizuseki, K., & Buzsaki, G. (2014). Theta oscillations decrease spike synchrony in the hippocampus and entorhinal cortex. Philosophical transactions of the Royal Society of London. *Series B. Biological sciences,**369*(1635), 20120530–20120530. 10.1098/rstb.2012.0530 PMC3866449 24366139

[CR13] Mizuseki, K., & Buzsáki, G. (2013). Preconfigured, skewed distribution of firing rates in the hippocampus and entorhinal cortex. *Cell Reports,**4*, 1010–1021. 23994479 10.1016/j.celrep.2013.07.039 PMC3804159

[CR14] Nigam, S., Shimono, M., Ito, S., Yeh, F.-C., Timme, N., Myroshnychenko, M., Lapish, C. C., Tosi, Z., Hottowy, P., Smith, W. C., Masmanidis, S. C., Litke, A. M., Sporns, O., & Beggs, J. M. (2016). Rich-club organization in effective connectivity among cortical neurons. *The Journal of Neuroscience,**36*(3), 670–684. 26791200 10.1523/JNEUROSCI.2177-15.2016 PMC4719009

[CR15] Okun, M., Steinmetz, N., Cossell, L., Iacaruso, M. F., Ko, H., Barthó, P., Moore, T., Hofer, S. B., Mrsic-Flogel, T. D., Carandini, M., & Harris, K. D. (2015). Diverse coupling of neurons to populations in sensory cortex. *Nature,**521*(7553), 511–515. 25849776 10.1038/nature14273 PMC4449271

[CR16] Ponce-Alvarez, A., Jouary, A., Privat, M., Deco, G., & Sumbre, G. (2018). Whole-brain neuronal activity displays crackling noise dynamics. *Neuron,**100*(6), 1446–14596. 30449656 10.1016/j.neuron.2018.10.045 PMC6307982

[CR17] Song, S., Sjöström, P. J., Reigl, M., Nelson, S., & Chklovskii, D. B. (2005). Highly nonrandom features of synaptic connectivity in local cortical circuits. *PLoS Biology,**3*(3), 68–68. 10.1371/journal.pbio.0030068 PMC1054880 15737062

[CR18] Stringer, C., Pachitariu, M., Steinmetz, N., Reddy, C. B., Carandini, M., & Harris, K. D. (2019). Spontaneous behaviors drive multidimensional, brainwide activity. *Science,**364*(6437), 255–255. 31000656 10.1126/science.aav7893 PMC6525101

[CR19] Vogel, R. M., Papalexiou, S. M., Lamontagne, J. R., & Dolan, F. C. (2025). When heavy tails disrupt statistical inference. *The American Statistician,**79*(2), 221–235.

[CR20] Wang, S.S.-H., Shultz, J. R., Burish, M. J., Harrison, K. H., Hof, P. R., Towns, L. C., Wagers, M. W., & Wyatt, K. D. (2008). Functional trade-offs in white matter axonal scaling. *The Journal of Neuroscience,**28*(15), 4047–4056. 18400904 10.1523/JNEUROSCI.5559-05.2008 PMC2779774

[CR21] Yassin, L., Benedetti, B. L., Jouhanneau, J.-S., Wen, J. A., Poulet, J. F. A., & Barth, A. L. (2010). An embedded subnetwork of highly active neurons in the neocortex. *Neuron,**68*(6), 1043–1050. 21172607 10.1016/j.neuron.2010.11.029 PMC3022325

[CR22] Zimek, A., & Filzmoser, P. (2018). There and back again: Outlier detection between statistical reasoning and data mining algorithms. Wiley interdisciplinary reviews. *Data mining and knowledge discovery,**8*(6), 1280.

